# Progressive Axonal Neuropathy and Cerebellar Ataxia Associated with Homozygous Pathogenic *POLG* Mutation in a Patient of African Descent from Northern Minas Gerais/Jequitinhonha Valley, Brazil: A Case Report and Epidemiological Implications

**DOI:** 10.5334/tohm.1238

**Published:** 2026-09-04

**Authors:** Caroline Fernandes Melo, Laura Toledo de Vasconcelos, Lays Aparecida Bono Evangelista

**Affiliations:** 1 Hospital Felício Rocho, Belo Horizonte, Minas Gerais, Brazil. CRM-MG 59750 | RQE 39684/55023; 2 Division of Medical Semiology and Neurology, Pontifícia Universidade Católica de Minas Gerais (PUC Minas), Belo Horizonte, Brazil; 3 MSc in Neurology, Universidade de São Paulo, Ribeirão Preto (USP-RP), Brazil

**Keywords:** mitochondrial disease, cerebellar ataxia, POLG, axonal neuropathy, consanguinity, health disparities, Brazil

## Abstract

We report a 41-year-old man of African descent from the Northern Minas Gerais/Jequitinhonha Valley region of Brazil — historically characterized by geographic isolation, predominantly Afro-Indigenous ancestry, and structural consanguinity — born to first-degree cousin parents, who presented with progressive axonal sensorimotor neuropathy, cerebellar ataxia, dysarthria, and oculomotor abnormalities. His sister had died with a similar untreated neurological syndrome. Three serial electroneuromyographic studies demonstrated stable chronic axonal polyneuropathy with complete bilateral absence of sural, fibular, and plantar sensory potentials, and active distal denervation. Brain MRI, unchanged over a two-year interval, showed T2/FLAIR hyperintense foci in both cerebellar hemispheres and the right middle cerebellar peduncle with cerebellar atrophy, without diffusion restriction or contrast enhancement. Next-generation sequencing (NGS; Mendelics®; 101-gene hereditary neuropathy panel) identified a homozygous pathogenic variant in the *POLG* gene (c.2243G>C; p.Trp748Ser — ClinVar ID 13507), confirming the diagnosis of mitochondrial DNA depletion syndrome type 4B (OMIM #613662). The patient’s paternal great-grandfather was from the state of Ceará, a region with documented European colonization, providing a plausible route for the introduction of this classically European founder allele into his Afro-Brazilian lineage, with homozygosity emerging through consanguinity. To our knowledge, this is the first report of p.Trp748Ser in a patient of African descent, directly challenging its presumed ethnic exclusivity. Clinical and electrophysiological stability over 2.5 years is consistent with the favorable course reported in p.W748S homozygotes. This case underscores the importance of *POLG*-related ataxia as a diagnostic consideration in patients of non-European ancestry presenting with axonal neuropathy and cerebellar signs, and highlights the consequences of underrepresentation of populations of African descent in genetic disease research.

## Introduction

Mitochondrial diseases associated with *POLG* gene mutations present a broad clinical spectrum, ranging from severe infantile hepatoencephalopathy to adult-onset ataxic neuropathy syndromes [1,2]. The *POLG* gene (chromosome 15q26.1) encodes the catalytic subunit of mitochondrial DNA (mtDNA) polymerase gamma, essential for mtDNA replication and repair. Over 300 pathogenic variants have been identified, causing at least six phenotypic spectra [3,5].

The ataxic neuropathy spectrum (ANS) — which encompasses the mitochondrial recessive ataxia syndrome (MIRAS) and the sensory ataxic neuropathy, dysarthria, and ophthalmoplegia (SANDO) syndromes — is the most common adult-onset presentation, characterized by the triad of cerebellar ataxia, sensory axonal neuropathy, and ophthalmoplegia, often accompanied by dysarthria, epilepsy, and cognitive impairment [4,6]. Epidemiological studies suggest that *POLG* mutations account for a significant proportion of recessive and sporadic ataxias in adults.

The p.Trp748Ser (c.2243G>C) variant is among the best-characterized pathogenic variants in *POLG*. Haplotype analysis has confirmed that all known carriers in European populations — including Finland, Norway, the United Kingdom, Belgium, Australia, and New Zealand — share a single ancient European founder chromosome [7,8]. No prior report has documented this variant in a patient of African descent. Critically, *POLG*-related disorders carry an absolute contraindication to valproic acid (VPA), with fatal outcomes documented in 52% of exposed patients with recessive *POLG* mutations [10].

We report the first case of the p.Trp748Ser variant in homozygosity in a patient of African descent and discuss the diagnostic, therapeutic, and epidemiological implications.

## Case Report

A 41-year-old man of African descent from the Northern Minas Gerais/Jequitinhonha Valley — a region characterized by geographic isolation, predominantly Afro-Indigenous ancestry, and high rates of consanguineous unions — was referred for progressive distal numbness, gait instability, and dysarthria over approximately one year. He described loss of plantar sensation, progressive slowing of cognition, and difficulty with fine motor coordination. He denied smoking; alcohol use was social. Medical history included hypertension, dyslipidemia, and an anxiety disorder, managed with enalapril, rosuvastatin, and paroxetine.

Notably, the anxiety disorder may not be incidental: affective and neuropsychiatric symptoms, including anxiety and depression, are increasingly recognized as part of the phenotypic spectrum of *POLG*-related disease, and may antedate or accompany the neurological manifestations [2].

Family history was highly informative: his parents were first-degree cousins (he was their eighth child), and a sister had died at age 47 following a progressive, undiagnosed neurological syndrome with phenotypic features identical to his own, ultimately wheelchair-dependent. His paternal great-grandfather was from the state of Ceará — a northeastern Brazilian state with documented European colonization and complex Afro-European genetic admixture since the 16th century — providing a plausible route for the introduction of a European founder allele into the family lineage. The pedigree is shown in Figure 2.

Neurological examination revealed cerebellar dysarthria, mild-to-moderate ataxic gait, areflexia in the lower limbs, preserved upper limb reflexes (2+/4+), and distal length-dependent sensory loss. No fasciculations, muscle atrophy, or pyramidal signs were present. Over the course of follow-up, slowing of ocular movements with fragmented saccades and left-sided sensorineural hearing loss emerged.

An extensive workup to exclude acquired and alternative hereditary causes was unremarkable. Serum lactate and vitamin B12 (473 pg/mL) were normal. A comprehensive immunological screen — including antinuclear antibodies (ANA), extractable nuclear antigen (ENA) antibodies, and anti-transglutaminase (coeliac) antibodies — was negative, as were the thrombophilia screen and urine porphobilinogen. Infectious serologies for HIV, hepatitis B and C, and toxoplasmosis were negative; IgG positivity for cytomegalovirus, Epstein–Barr virus, herpes simplex virus types 1 and 2, and varicella-zoster virus indicated past exposure without evidence of active infection. Notably, genetic testing for hereditary transthyretin (TTR) amyloidosis — a key differential for progressive axonal sensorimotor neuropathy — was negative; this same next-generation sequencing investigation subsequently identified the causative POLG variant (see below).

Three serial electroneuromyographic studies were performed. The first study (lower limbs) demonstrated complete absence of sensory action potentials in the bilateral sural, superficial fibular, and medial plantar nerves, with preserved motor conduction and F-waves. Needle EMG revealed chronic denervation-reinnervation with large-amplitude, long-duration motor unit potentials, reduced recruitment, and sparse spontaneous activity (positive sharp waves) in distal lower limb muscles. This coexistence of chronic reinnervation with sparse active denervation reflects an ongoing, slowly progressive axonal process in which continued denervation is only partially compensated by reinnervation — a pattern consistent with the chronic but active neuropathy of *POLG*-related disease. The second study (upper limbs) showed mildly reduced sensory amplitudes in median, ulnar, and radial nerves bilaterally with normal conduction velocities and fully preserved motor conduction. The third study (four limbs) confirmed stability without significant electrophysiological progression over seven months.

**Figure 1:**
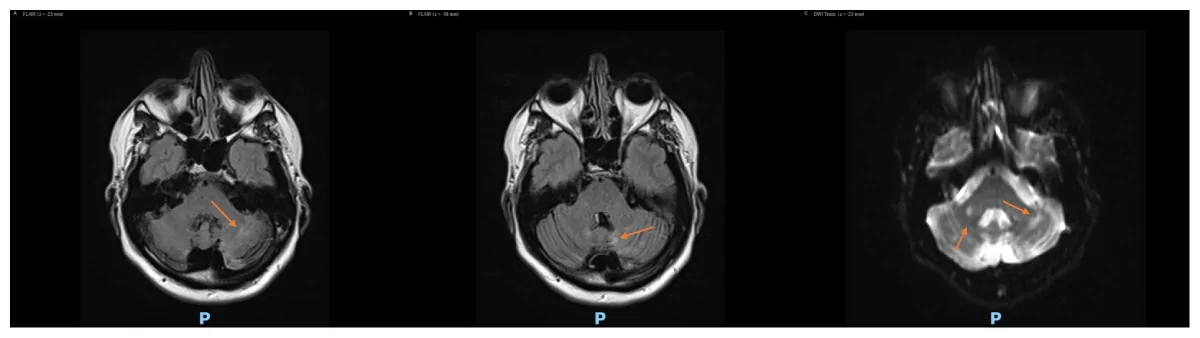
Brain MRI. **(A)** and **(B)**: Axial FLAIR sequence at two infratentorial levels showing small T2/FLAIR hyperintense foci in both cerebellar hemispheres and the right middle cerebellar peduncle (red arrows), with cerebellar atrophy. **(C)**: Diffusion-weighted imaging (DWI Trace) at the same level demonstrating absence of diffusion restriction, excluding acute ischemic etiology. Findings were stable over a 26-month interval. Images fully deidentified with written patient consent.

Brain MRI with gadolinium demonstrated small T2/FLAIR hyperintense foci in both cerebellar hemispheres and the right middle cerebellar peduncle, hypointense on T1, without diffusion restriction or contrast enhancement, associated with cerebellar atrophy (widening of the posterior fossa cerebrospinal fluid spaces). The supratentorial parenchyma was preserved. A follow-up study confirmed stability of these findings over a 26-month interval (Figure 1). The imaging was archived as an axial-only protocol; a dedicated sagittal sequence was not available, which constitutes a minor limitation of the retrospective imaging documentation.

**Figure 2:**
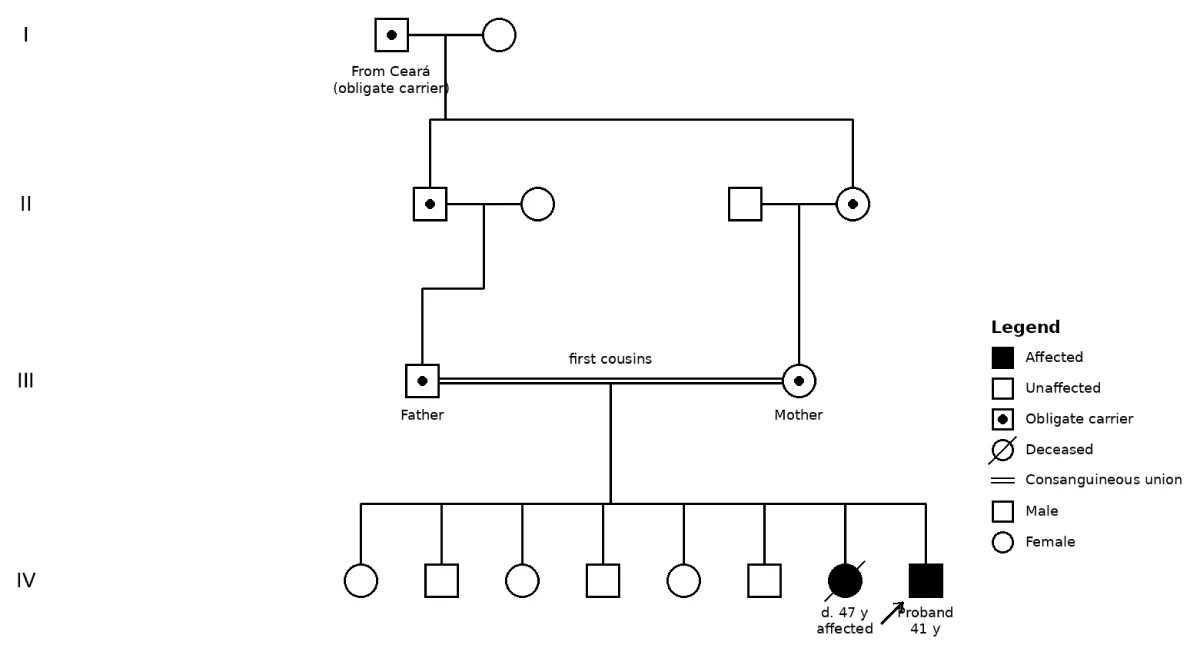
Pedigree of the family. The proband (arrow, generation IV) is an affected male; an affected sister died at age 47. Parents are first-degree cousins (consanguineous union, double line). The paternal great-grandfather from Ceará (generation I) is the presumed obligate carrier through whom the European founder allele entered the lineage. Squares = males; circles = females; filled symbols = affected; central dot = obligate carrier; diagonal line = deceased.

In light of the progressive axonal neuropathy, cerebellar signs, oculomotor abnormalities, and family history consistent with autosomal recessive inheritance in a consanguineous setting, a 101-gene hereditary neuropathy NGS panel was requested (Mendelics Análise Genômica, São Paulo). Technical quality was excellent: 100% target base coverage ≥ 10 reads, mean depth 87×, 28,860,918 sequences generated. A homozygous pathogenic variant was identified in *POLG*: c.2243G>C (chr15:89,323,426; GRCh38), p.Trp748Ser (ClinVar ID 13507 [12], pathogenic score 5/5), present in heterozygosity in only 124/~141,000 individuals in gnomAD and extremely rare in homozygosity (Table 1).

**Table 1:** Summary of genetic and molecular findings (Mendelics®, BRP448-001).


PARAMETER	RESULT

Laboratory	Mendelics Análise Genômica (CRM-SP 955.471), São Paulo, Brazil

Test/Code	Hereditary Neuropathy Panel — BRP448-001 (101 genes)

Method	NGS (Illumina) — probe-based target capture; alignment to GRCh38

Quality metrics	100% target bases ≥ 10 reads; mean depth 87×; 28,860,918 sequences generated

Gene (OMIM)	POLG — DNA Polymerase Gamma, Catalytic Subunit (OMIM* 174763)

Genomic position	chr15:89,323,426 (GRCh38)

cDNA variant	c.2243G>C (ENST00000268124)

Protein change	p.Trp748Ser

Zygosity	Homozygous (2 copies)

ClinVar classification	Pathogenic — Variation ID 13507; score 5/5

Population frequency	Heterozygous in 124/~141,000 individuals (gnomAD); homozygosity extremely rare

Molecular diagnosis	Mitochondrial DNA depletion syndrome type 4B (OMIM #613662)

Release date	July 20, 2020


The definitive diagnosis of mitochondrial DNA depletion syndrome type 4B (OMIM #613662) was established. Mitochondrial supportive therapy with coenzyme Q10 was initiated. During 2.5 years of follow-up, the patient showed gradual progression of cerebellar ataxia and scanning dysarthria but maintained independent ambulation throughout. Valproic acid was never prescribed; the patient and family were counseled regarding its absolute contraindication.

## Discussion

*POLG*-related diseases represent one of the most heterogeneous groups within mitochondrial encephalomyopathies [2]. The present case illustrates the ANS/SANDO phenotype in a young adult: progressive axonal sensorimotor neuropathy with cerebellar ataxia, dysarthria, and oculomotor involvement in an autosomal recessive consanguineous setting — fully concordant with established diagnostic criteria [4,6].

The most significant contribution of this report is the identification of the p.Trp748Ser variant in homozygosity in a patient of African descent without documented European ancestry. This variant is one of the most emblematic European founder mutations in rare neurological disease: Hakonen et al. demonstrated by haplotype analysis that all known carrier chromosomes across Finland, Norway, the United Kingdom, Belgium, Australia, and New Zealand trace back to a single ancient European ancestor [8]. To our knowledge, no prior publication has reported this variant — in any zygosity — in a patient of African descent (Table 2).

**Table 2:** Comparison of the present case with published cases of POLG disease harboring the p.Trp748Ser variant.


REFERENCE	POLG GENOTYPE	ONSET (YRS)	AXONAL NEUROPATHY	ATAXIA	OPHTHALMOPLEGIA	EPILEPSY	OTHER FINDINGS	OUTCOME

Hakonen et al., 2005	p.W748S/p.E1143G hom.	20–40	Yes (sensory)	Yes	Yes	Common	Dysarthria; Finnish ancestry	Progressive

Hakonen et al., 2007	p.W748S hom. — multiple European populations	15–45	Yes (sensory)	Yes	Yes	Common	Single European founder haplotype confirmed	Progressive

Tzoulis et al., 2006	p.W748S hom. vs A467T/W748S het. comp. (n = 26)	Median 30	Yes	Yes	Yes (late)	66%	Longer survival in homozyg. (p = 0.006)	Progressive

Van Goethem et al., 2004	p.W748S + other variants	Adult	Yes (axonal)	Yes, cerebellar	Yes	Yes	No myopathy	Progressive

Nuzhnyi et al., 2021	p.W748S hom. 72.7%; Russian cohort (n = 11)	30–50	Yes (axonal)	Yes	Some patients	Rare	Consanguinity in 3/11	Progressive

Present case (Brazil)	p.W748S hom. (c.2243G>C)	40	Yes (sensorimotor axonal)	Yes, cerebellar	Fragmented saccades	No	Patient of African descent; N. Minas Gerais/Jequitinhonha Valley; great-grandfather from Ceará; first-degree consanguinity; sensorineural hearing loss; first report in a patient of African descent	Independent ambulation; stable over 2.5 years


hom. = homozygous; het. comp. = compound heterozygous; NGS = next-generation sequencing.

The identification of the patient’s paternal great-grandfather as a native of Ceará — a Brazilian state with a complex colonial history of Afro-European admixture — provides the most parsimonious biological explanation: the European founder allele likely entered the family lineage through this ancestor in heterozygous state, circulating silently across generations and reaching homozygosity in the proband through first-degree parental consanguinity. This mechanism mirrors other founder allele dynamics described in admixed Afro-Brazilian populations and exemplifies how pathogenic variants of European origin can propagate cryptically in non-European lineages [8].

Electrophysiological stability over seven months and imaging stability over 26 months are consistent with the slowly progressive course documented by Tzoulis et al. in p.W748S homozygotes, who showed significantly longer survival compared to compound heterozygotes (p = 0.006) [9]. The present patient maintained independent ambulation throughout 2.5 years of follow-up, further supporting the relatively favorable prognosis of this genotype.

From a pharmacological standpoint, the diagnosis permitted avoidance of VPA — a critical outcome given the 52% fatality rate documented in *POLG*-mutation carriers exposed to valproate in a 2023 systematic review [10,11]. The scoping review by Kalampokini et al. (2025) — published in this journal — reinforces that *POLG*-related cerebellar ataxia should be systematically included in the differential diagnosis of slowly progressive ataxias, regardless of the patient’s ethnic background [13].

This case also highlights a broader concern in neurogenetics: the underrepresentation of populations of African descent and admixed populations in rare disease genetic research. The presumption that p.Trp748Ser is exclusively European could lead to its omission from diagnostic panels in non-European patients with compatible phenotypes, perpetuating diagnostic invisibility. Our report challenges this assumption and supports expanding *POLG* screening to patients of any ethnic background presenting with axonal neuropathy and cerebellar signs, particularly in consanguineous settings.

## Conclusions

We report the first known case of the *POLG* p.Trp748Ser variant in homozygosity in a patient of African descent, from the Northern Minas Gerais/Jequitinhonha Valley, Brazil. The probable route of allele introduction via an admixed great-grandfather from Ceará, amplified to homozygosity by consanguinity, exemplifies how European founder variants can manifest in non-European populations. This case underscores the importance of early *POLG* genetic testing in patients with axonal neuropathy and cerebellar signs regardless of ethnic background, and reinforces the clinical urgency of the diagnosis to prevent fatal valproic acid exposure.
